# Pompe practice survey of Canadian (Ontario) rheumatologists

**DOI:** 10.1186/1471-2474-14-S2-P4

**Published:** 2013-05-29

**Authors:** Carter Thorne, Mark Tarnopolsky

**Affiliations:** 1 McMaster University, Hamilton, Ontario, Canada

## Introduction

Pompe disease (glycogenosis II, acid maltase deficiency, OMIM 232300) is a treatable autosomal recessive disorder of glycogen metabolism caused by deficiency of the lysosomal enzyme acid alpha-glucosidase. A hallmark of Pompe disease is the presence of glycogen-loaded lysosomes. Pompe disease has frequently been misdiagnosed as other myopathies, such as polymyositis, and mistakenly treated with steroids. A rheumatology based practice survey is being designed to establish the incidence of Pompe disease and other myopathies in patients with atypical steroid-unresponsive presumed myopathies (ASUPM).

## Methods

Five to ten self-identified rheumatologists with an interest in myopathies and ‘community’ practices located in Ontario, Canada, will review medical records to identify patients with ASUPM. A standardized chart review tool to help expedite the review will be developed and approved by the participants. Charts of any patient seen in the past five years with a diagnostic code of 710, 729, 739 or 781 will be identified and data extracted using the standardized CRF; data extraction will be performed by the physician or a trained delegate. Central IRB approval will be obtained, and participants will be eligible for CPD credits. Physicians will also provide demographic information (i.e., years in practice, practice profile, total charts reviewed, etc.), and patient data will include, but is not limited to the following: CK values, serology findings, biopsy results, steroid responsiveness, and diagnoses (e.g., polymyositis, dermatomyositis). Identified patients will be referred to a single neuromuscular practitioner (Mark Tarnopolsky) who will perform dried blood spot, enzymatic, and genetic tests to determine the exact nature of the myopathy and the incidence and prevalence of these steroid non-responsive myopathies in the ASUPM population.

## Discussion

Presentation at the 6th Annual European Symposium will provide an opportunity for us to further develop this project based on expert feedback. Our overarching goals are to: 1) develop strategies/tools to facilitate efficient, systematic chart review; 2) ascertain cases of atypical myopathy/myositis or other unclear diagnoses that may be Pompe disease; 3) improve physician awareness of uncommon and rare diagnoses; and 4) share the results with a wider rheumatology community.

